# Integrated Multi-Omics Exploration of the Molecular Mechanisms Underlying Differential Responses to Saline-Alkaline Stress in Leaves of *Populus simonii* × *P. nigra*

**DOI:** 10.3390/biology15181656

**Published:** 2026-09-18

**Authors:** Xue Leng, Sisu Li, Jingli Yang, Shicheng Zhao, Jinhui Gao, Ke Ma, Hanzeng Wang

**Affiliations:** 1 College of Agriculture, Jilin Agricultural Science and Technology College, Jilin 132101, China; lengxue@jlnku.edu.cn (X.L.); 15590095105@163.com (S.L.); 2 State Key Laboratory of Tree Genetics and Breeding, Northeast Forestry University, Harbin 150040, China; yifan85831647@nefu.edu.cn; 3 School of Pharmacy, Harbin University of Commerce, Harbin 150040, China; zhaoshicheng@msn.com; 4 Heilongjiang Academy of Forestry Yichun Branch, Yichun 153000, China; zhl737603@163.com

**Keywords:** *Populus simonii* × *P. nigra*, leaves, alkaline salt stress, transcriptome, metabolome

## Abstract

*Populus simonii* × *P. nigra* is a valuable model for studying woody plant responses to saline-alkaline stress, however the molecular mechanisms underlying its adaptation remain poorly understood. Here, integrated transcriptomic and metabolomic analyses were used to examine the responses in leaves to low (50 mmol/L) and high (200 mmol/L) alkaline salt stress over 7 and 14 days. Physiological assessments revealed that high-concentration stress inhibited growth, induced oxidative damage, elevated malondialdehyde (MDA) content, enhanced antioxidant enzyme activity (SOD, POD, CAT), and promoted osmoregulatory compound accumulation (soluble sugars, proline, free amino acids). RNA sequencing revealed extensive transcriptional reprogramming, with more pronounced changes under higher stress and longer exposure. KEGG enrichment highlighted pathways such as plant hormone signal transduction starch/sucrose metabolism, and flavonoid and phenylpropanoid biosynthesis. Metabolomic profiling detected 4257 metabolites, with notable increases in amino acids, organic acids, sugars, and flavonoids. Integrated analyses revealed coordinated regulation of genes and metabolites in phenylalanine, tyrosine, and tryptophan biosynthesis, as well as hormone signaling. Weighted gene co-expression network analysis (WGCNA) identified modules correlated with physiological traits, enriched in stress-responsive transcription factors (MYB, AP2/ERF, NAC, WRKY) and metabolic pathways. Key genes in starch/sucrose degradation, and raffinose metabolism were upregulated, whereas phenylpropanoid metabolism-related genes were suppressed. Collectively, these findings provide insight into the adaptive mechanisms of *P. simonii* × *P. nigra* to saline-alkaline stress, highlighting regulatory genes and pathways that contribute to tolerance and offering potential targets for enhancing poplar resilience in alkaline salt environments.

## Simple Summary

Global soil salinization poses a severe threat to plant survival. This study aims to explore how *Populus simonii* × *Populus nigra*, a hybrid poplar species widely used for afforestation, adapts to harsh environments. The results demonstrated that under high-concentration saline-alkaline stress, the poplar modulates internal gene activities to produce and accumulate large amounts of protective sugars, amino acids, and antioxidant components, thereby maintaining water balance and protecting plant cells. By revealing the survival mechanisms of this species and identifying core regulatory genes, this study provides an essential blueprint for breeding new stress-resistant tree varieties in the future, offering significant value for restoring degraded soils and improving ecological environments.

## 1. Introduction

Soil salinization is a major global environmental challenge, affecting approximately 8.31 × 10^8^ hectares of land and continuing to expand [1]. Compared with salt stress alone, saline-alkaline stress is more severe because it combines high sodium toxicity, osmotic stress, and elevated pH resulting from increased soil alkalinity [2]. Under these conditions, plants must contend with ionic toxicity, osmotic imbalance, oxidative damage, and nutrient deficiencies caused by the hydrolysis of NaHCO_3_ and Na_2_CO_3_. Saline-alkaline stress also disrupts ion homeostasis, damages cellular membranes, impairs osmotic adjustment, and severely compromises morphogenesis. In particular, alkali stress dominated by alkaline salts directly interferes with cellular functions and strongly inhibits plant growth and development [3].

To withstand saline-alkali stress, plants have evolved multilayered adaptive mechanisms involving gene regulation, protein turnover, and metabolic adjustment [4]. In addition, plants employ an integrated stress response system that coordinates osmotic regulation, ion homeostasis, and antioxidant defenses. Key strategies include synthesizing compatible solutes to balance osmotic pressure, excluding or compartmentalizing excess sodium through ion transporters, and activating antioxidant enzymes to mitigate oxidative damage [5]. Sustained sodium accumulation in saline-alkali soils raises extracellular osmotic pressure, driving water efflux and causing physiological drought. To counter this, plant cells accumulate low-molecular-weight osmolytes such as proline, betaine, soluble sugars, polyols, polyamines, and soluble proteins [6]. These compounds help stabilize water potential, maintain osmotic balance, protect proteins, and preserve macromolecular structure [5]. For example, transgenic tobacco plants overexpressing *PtWRKY39* accumulate higher levels of proline and soluble proteins and exhibit increased chlorophyll content, reduced MDA accumulation and lower ROS levels [7]. These changes improve membrane stability and osmotic adjustment capacity, thereby markedly enhancing stress tolerance [7]. Similarly, wheat exposed to saline-alkali stress shows elevated proline, soluble sugars, and polyols such as sorbitol, underscoring the critical role of osmotic adjustment in plant adaptation to saline-alkaline stress [8].

Under saline-alkaline stress, plants rapidly accumulate ROS such as superoxide anion (O_2_·^−^), hydroxyl radical (OH·), hydrogen peroxide (H_2_O_2_), singlet oxygen (^1^O_2_), and ozone (O_3_). Although ROS function as important signaling molecules during stress responses, their excessive accumulation can cause severe oxidative damage To mitigate toxicity, plants employ a dual antioxidant defense system, comprising enzymatic antioxidants, such as peroxidase (POD), superoxide dismutase (SOD), and catalase (CAT), as well as non-enzymatic antioxidants such as ascorbic acid, glutathione, and flavonoids [9]. Functional studies highlight the importance of ROS detoxification in stress tolerance. For example, the transcription factor (TF) ChbZIP1 from alkaliphilic microalga *Chlorella* sp. BLD enhances alkaline tolerance in *Arabidopsis thaliana* by activating ROS-scavenging pathways [10]. Similarly, SlWRKY28 from *Tamarix chinensis* improves saline-alkali tolerance in poplar by inducing ROS-scavenging enzymes [11]. These findings suggest that different species, and even varieties within a species, employ distinct strategies, often relying on specific osmolytes or antioxidant pathways. Despite advances in *Arabidopsis* [12] and some crops [13], research on combined saline-alkaline (soda saline) stress, is still limited. To date, only a few related genes have been identified, and the adaptive mechanisms of perennial woody plants remain poorly understood.

When exposed to abiotic stresses such as drought, salinity, extreme temperatures, and UV radiation, plants activate the phenylpropanoid biosynthesis pathway, leading to the increased synthesis and accumulation of diverse phenolic compounds [14]. This pathway underlies the production of more than 8000 metabolites, including flavonoids, lignin, and their derivatives, making it a critical target for breeding climate-resilient crops [15]. Phenylalanine ammonia-lyase (PAL), the first committed enzyme, converts phenylalanine into trans-cinnamic acid. Cinnamate 4-hydroxylase (C4H), the first of three cytochrome P450 enzymes involved in lignin biosynthesis, then hydroxylates this intermediate to form 4-coumaric acid. Next, 4-coumarate-CoA ligase (4CL) generates a CoA-thioester and ultimately p-coumaroyl-CoA to catalyzes the formation of p-coumaroyl-CoA, a central hub that branches into multiple downstream pathways producing lignans, flavonoids, hydroxycinnamic esters, salicylic acid, tannins, and related metabolites [16,17].

Flavonoid biosynthesis directly to the phenylpropanoid pathway is one of the major downstream branches of this pathway, generating numerous physiologically active secondary metabolites [18]. Under high-pH stress, plants also accumulate and secrete organic acids to buffer the rhizosphere and maintain ion homeostasis [19]. In alfalfa (*Medicago sativa*), pathways such as phenylpropanoid and flavonoid biosynthesis, starch and sucrose metabolism, and plant hormone signal transduction play central roles in the saline-alkaline stress response [20]. Under saline-alkali stress, the MsMYB12 TF directly regulates MsFLS13 expression, driving flavonol accumulation and enhancing antioxidant capacity. The alfalfa cultivar ‘Zhaodong’ shows stronger regulation of flavonoid metabolism and greater stress tolerance than ‘Blue Moon’ [21]. Similarly, in *Glycyrrhiza uralensis*, alkaline stress activates genes involved in flavonoid biosynthesis, promoting the accumulation of antioxidants such as 2′-hydroxygenistein and apigenin [22]. In rose petals, saline-alkali stress induces both flavonoid and terpenoid metabolism, with the TF MYB5 coordinating the biosynthesis of sesquiterpenes and proanthocyanidins by directly regulating the expression of *TPS31* and *ANR* [23].

Beyond secondary metabolism, the reprogramming of primary carbohydrate metabolism, particularly starch and sucrose metabolism, is a fundamental adaptive strategy against severe abiotic stress [24]. Under saline-alkaline conditions, the rapid degradation of starch yields soluble sugars such as sucrose, glucose, and fructose, which act as vital osmoprotectants to maintain cellular turgor and stabilize macromolecular structures. Furthermore, sucrose serves as the foundational precursor for the raffinose biosynthesis pathway. The accumulation of raffinose family oligosaccharides (RFOs) is a hallmark stress response in many plant species [25]. Raffinose not only functions as a compatible solute for osmotic adjustment but also acts as a potent non-enzymatic antioxidant, directly scavenging free radicals to protect chloroplasts and cellular membranes from abiotic-stress-induced oxidative damage [26,27]. Therefore, elucidating the transcriptional and metabolic dynamics of these specific carbohydrate pathways is crucial for comprehensively understanding the integrated stress tolerance mechanisms.

The Chinese Songnen Plain is one of the world’s three largest soda saline-alkali regions, where soil degradation is driven mainly by excess NaHCO_3_ and Na_2_CO_3_ [28]. In these natural habitats, plants are rarely subjected to a single type of stress; instead, they simultaneously face a complex combination of ionic toxicity, osmotic imbalance, and elevated pH [29]. *Populus simonii* × *P. nigra*, a widely afforested species in this region, exhibits notable tolerance to this harsh environmental conditions, making it a valuable genetic resource. While previous controlled studies have often isolated salt or pH stress to dissect specific molecular pathways, plants in the Songnen Plain endure these stresses concurrently.

Therefore, simulating this naturally occurring combined saline-alkaline stress is crucial for understanding the integrated and synergistic defense mechanisms of this species. We hypothesized that the robust saline-alkaline tolerance of *P. simonii* × *P. nigra* is governed by a hierarchical regulatory network, wherein stress-induced hormonal signaling activates specific master transcription factors to orchestrate targeted metabolic reprogramming. Specifically, this involves the prioritized accumulation of key carbohydrate osmoprotectants, including sucrose and raffinose, alongside secondary antioxidant metabolites such as flavonoids, to synergistically counteract osmotic shock and oxidative damage. To test this hypothesis, we utilized a mixture of NaHCO_3_ and Na_2_CO_3_ to mimic native soil conditions and investigated the effects of combined saline-alkaline stress at different concentrations and exposure durations on the leaves of *P. simonii* × *P. nigra* through integrated transcriptomic and metabolomic analyses. Physiological parameters, including soluble protein, soluble sugar, MDA, amino acid, and proline contents, as well as the activities of SOD, CAT, and POD, were measured to assess the phenotypic and biochemical validity of this adaptive response. Weighted gene co-expression network analysis (WGCNA) further revealed the metabolic pathways and regulatory networks activated under stress. Together, these findings provide a comprehensive view of the molecular and metabolic adaptations that support the saline-alkaline stress of *P. simonii* × *P. nigra*.

## 2. Materials and Methods

### 2.1. Plant Growth Conditions and Stress Treatments

To assess the effects of saline-alkaline stress on *P. simonii* × *P. nigra*, two-year-old branch cuttings were collected from Jilin Agricultural Science and Technology College in April 2024. Cuttings were trimmed to 10 cm in length and transplanted into uniform plastic pots (15 cm diameter and 13.2 cm height). Each pot was filled with exactly 400 g of uniformly mixed substrate containing humus, vermiculite, and perlite (5:3:2, *v*/*v*/*v*). Plants were grown in a greenhouse at 24 ± 2 °C under a 16 h light/8 h dark photoperiod 5000 lx photoperiod and irrigated twice weekly with 250 mL of Hoagland’s nutrient solution. After six weeks, uniformly developed seedlings were selected for stress treatments. Alkaline solutions were prepared by dissolving equimolar amounts of Na_2_CO_3_ and NaHCO_3_ in Hoagland’s solution at final concentrations of 0 (control), 50 (low stress), and 200 mmol/L (high stress). These concentrations were selected to mimic the heterogeneous ecological conditions of the Songnen Plain, a typical soda saline-alkali region [20]. The 50 mM concentration represents mild-to-moderate saline-alkaline stress and is widely used to evaluate stress adaptation in species such as alfalfa and wild soybean [28]. For severe stress, our preliminary experiments indicated that a 250 mM concentration caused complete seedling mortality within 5 days, precluding long-term physiological observations. Thus, the 200 mM concentration was selected as the highest sublethal concentration to reflect the severe saline-alkaline patches found in heavily degraded soils. Given that *P. simonii* × *P. nigra* is an important pioneer tree species used for afforestation species in this region, this design provides a comprehensive evaluation of its adaptive mechanisms up to its extreme survival limit. Plants were irrigated with 250 mL of the respective solutions twice weekly. Each treatment consisted of three independent biological replicates, with three individual plants per replicate, totaling nine plants per treatment group. Leaves were sampled on days 7 and 14, immediately frozen in liquid nitrogen, and stored at −80 °C until subsequent physiological, transcriptomic, and metabolomic analyses.

### 2.2. RNA Extraction, Sequencing, and Differential Gene Expression

Total RNA was extracted using an RNAprep Pure Plant Plus Kit (Tiangen, Beijing, China), and integrity was assessed with a NanoDrop 2000 spectrophotometer (Thermo Fisher Scientific, Waltham, MA, USA). cDNA libraries were prepared with a VAHTS Universal V5 RNA-seq Library Prep Kit (Vazyme Biotech Co., Ltd., Nanjing, China) and sequenced on the Illumina Hiseq 2000 platform by Beijing Biomarker Technologies Corporation (Beijing, China). Differentially expressed genes (DEGs) were identified by comparing the CK-7d and CK-14d controls with L-7d, H-7d, L-14d, and H-14d treatments. Raw sequencing data were quality-filtered to remove adapters, poly-N reads, and low-quality reads. The Populus trichocarpa genome (v4.1) was utilized as the reference due to its high-quality annotation and the extensive genomic synteny conserved across the Populus genus. High-quality clean reads were aligned to this reference genome using HISAT2 (v2.2.1), and raw gene read counts were extracted using featureCounts (v1.5.0). Differential expression analysis was performed directly on the raw count data using the DESeq2 R package (v 1.50.2), with thresholds of |log_2_FC| > 1 and false discovery rate (FDR) < 0.01 [30]. Fragments per kilobase of transcript per million mapped reads (FPKM) were calculated solely to standardize expression levels for sample correlation and visualization.

For functional annotation, the identified genes were annotated by comparison with major public databases. Specifically, sequence alignments were performed using DIAMOND against the NCBI non-redundant protein sequences (Nr), UniProt [31], Kyoto Encyclopedia of Genes and Genomes (KEGG) (Release 116.0) [32], and Swiss-Prot [33]. Protein domain annotations were conducted using HMMER against the Pfam database (version 36.0) [34], while Gene Ontology (GO) [35] annotations were obtained using InterProScan 5. Sample consistency was evaluated using Pearson correlation coefficients from the FPKM matrix. DEGs were analyzed through volcano plots, hierarchical clustering, and functional enrichment. GO term and KEGG pathway enrichment analyses were performed using clusterProfiler (v3.14.3) [36], and DEG expression patterns were visualized with TBtools-II heatmaps (v 2.142) [37].

### 2.3. Metabolomic Analysis

Leaf samples were collected after 7 and 14 days of treatment, with water-treated plants serving as the controls. Metabolites were extracted by solid-phase extraction and analyzed using liquid chromatography–tandem mass spectrometry (LC–MS/MS). Leaf tissues were ground at 30 Hz for 1 min and stored at –80 °C. For each sample, 50 mg of powder was mixed with 10 μL of a 100 ng mL^−1^ internal standard and 1 mL of extraction solvent (methanol/water/formic acid, 15:4:1, *v*/*v*/*v*) then vortexed for 10 min and centrifuged at 12,000× *g* for 5 min at 4 °C. The supernatant was collected, concentrated, and reconstituted in 10 μL of 80% methanol. After filtration through a 0.22 μm membrane filter, the solution was transferred to injection vials for LC-MS/MS analysis. Metabolite identification was primarily achieved at MSI Level 2 (putatively annotated compounds) by matching accurate precursor mass (*m*/*z*), retention time, and MS/MS fragmentation spectra against an extensively curated in-house database constructed from authentic standards, supplemented by standard public metabolomics databases. Differentially accumulated metabolites (DAMs) were identified using thresholds of variable importance in projection (VIP) > 1.0, FC > 1.5 or <0.667, and *p* < 0.05. This combined approach, anchored by the multivariate OPLS-DA VIP score, was explicitly chosen to effectively control false positives while preventing the excessive false negatives (Type II errors) often associated with strict multiple-testing corrections in highly collinear untargeted metabolomics datasets.

### 2.4. Conjoint Analysis of Transcriptome and Metabolome Data

A bidirectional orthogonal partial least squares (O2PLS) model was used to integrate the transcriptomic and metabolomic datasets, with the aim of elucidating the systemic relationships between gene expression and metabolic remodeling in *P. simonii* × *P. nigra* under saline-alkali stress. Model reliability and predictive performance were evaluated using 7-fold cross-validation, with the coefficient of determination (R^2^) and predictive ability (Q^2^) as key metrics. DEGs and DAMs were identified based on VIP expand values, highlighting components critical for the response to different saline-alkaline levels.

### 2.5. Weighted Gene Co-Expression Network Analysis (WGCNA)

Gene co-expression networks were constructed using WGCNA (v1.47) in R (v3.5.1), after excluding genes with FPKM < 10. Gene co-expression modules were identified on the Biocloud platform (https://international.biocloud.net/) (accessed on 2 April 2025) using the blockwiseModules function. To ensure a scale-free network topology, an optimal soft-thresholding power (β) of 8 was selected. Modules were constructed with a minimum module size of 30 genes, and modules with highly similar expression profiles were subsequently merged using a mergeCutHeight of 0.25 (corresponding to a correlation threshold of 0.75). Module eigengenes were calculated and correlated with metabolite profiles, and the resulting networks were visualized using Cytoscape (v3.10.3).

### 2.6. Physiological Characterization and Enzyme Activities

Soluble protein content was determined using the Bradford method [38], and soluble sugar content by anthrone–sulfuric acid assay. MDA was quantified using the thiobarbituric acid method [39]. Free amino acids were determined by ninhydrin colorimetric assay, and proline using the acid-ninhydrin method [39]. The activities of SOD, POD, and CAT were determined using commercial assay kits (Suzhou Comin Biotechnology Co., Ltd., Suzhou, China). Specifically, SOD activity was determined based on its ability to inhibit the photochemical reduction of nitroblue tetrazolium (NBT). POD activity was measured colorimetrically based on the catalytic oxidation of guaiacol in the presence of H_2_O_2_ and CAT activity was quantified by directly monitoring the decomposition rate of H_2_O_2_ at 240 nm [39].

### 2.7. RT-qPCR Validation of DEGs

To validate the transcriptome sequencing results. RT-qPCR was performed on nine randomly selected DEGs. The *PsnActin* gene (GenBank accession no: XM_002298946) served as the internal reference [39,40]. Leaf samples collected from the low- and high-saline-alkaline stress treatments, identical to those used for RNA sequencing, were used for RNA extraction. First-strand cDNA was synthesized with 1 μg of total RNA using HiScript III RT SuperMix (Vazyme Biotech, Nanjing, China) according to the manufacturer’s instructions. Gene-specific primers were designed using Primer-BLAST (https://www.ncbi.nlm.nih.gov/tools/primer-blast) (accessed on 13 June 2025). An amount of 20 μL PCR reaction contained 10 μL of ChamQ SYBR Color Master Mix (Vazyme, Beijing, China), 2 μL of cDNA, 0.4 μL each of forward and reverse primers (10 μM), and 7.2 μL nuclease-free water. The thermocycling conditions were as follows: 95 °C for 7 min followed by 35 cycles of 95 °C for 15 s, 60 °C for 20 s, and 72 °C for 30 s. Amplification was performed using a qTOWER 3G Real-Time PCR System. (Analytik, Jena, Germany). Relative expression levels were calculated using the 2^−ΔΔCt^ method and visualized with GraphPad Prism v9.0.0 (GraphPad Software Inc., Boston, MA, USA) [41]. The sequences of the gene-specific primers are listed in Table S1.

### 2.8. Statistical Analysis

Statistical analysis was performed using SPSS software (versions 20.0 and 22.0; IBM, Armonk, NY, USA). Two-tailed Student’s *t*-test was used to evaluate the statistical significance of RT-qPCR data. One-way ANOVA followed by Duncan’s multiple comparison test was applied to analyze significant differences in growth traits and physiological indicators. Data are presented as the mean ± standard deviation (SD) of three biological replicates.

## 3. Results

### 3.1. Physiological Responses of P. simonii × P. nigra Leaves Under High and Low Saline-Alkaline Stress

**Figure 1:**
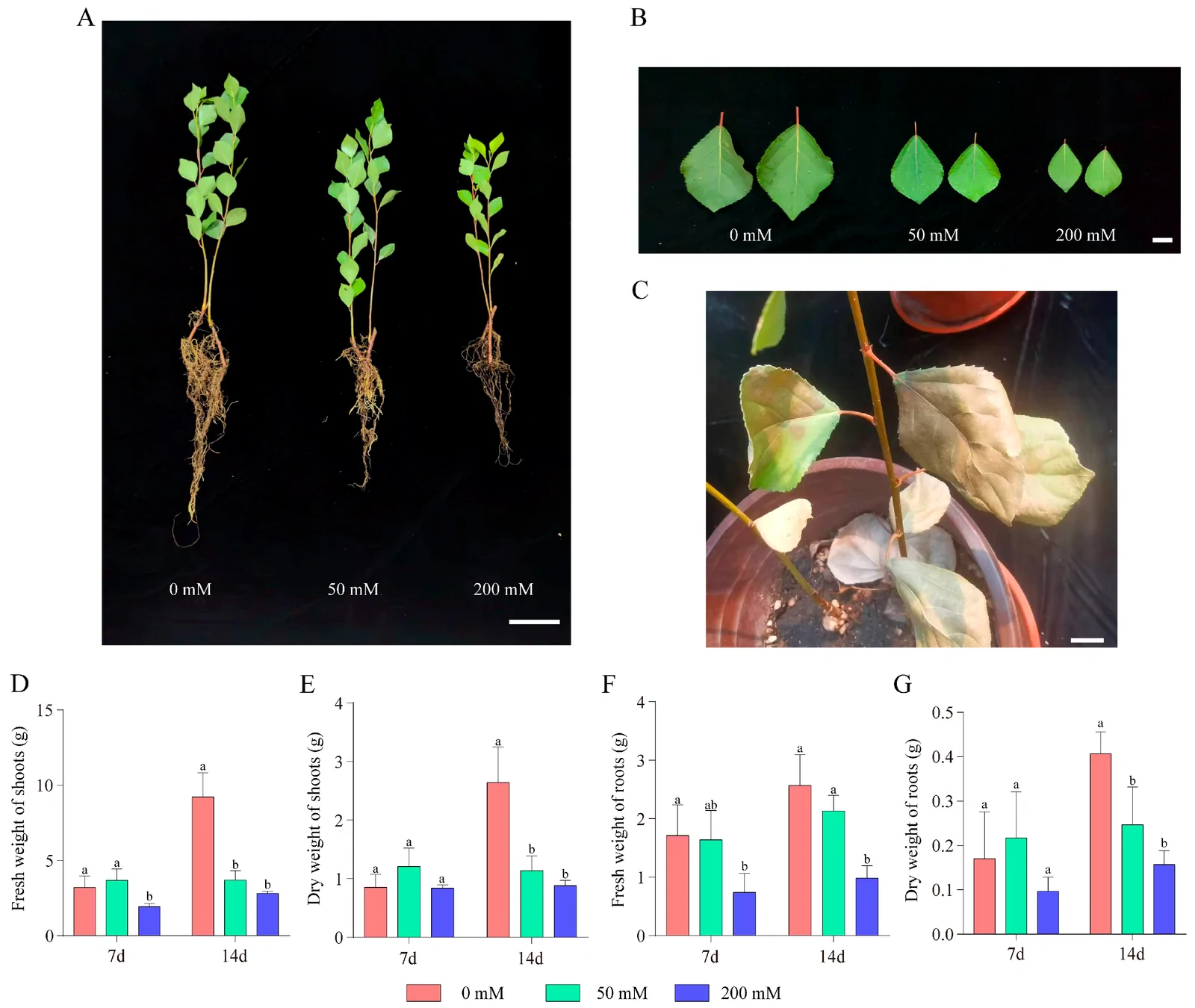
Effects of high and low concentrations saline-alkaline stress on growth and physiology of *P. simonii* × *P. nigra*. (**A**) Representative images of plants treated with alkaline salt stress for 14 days. Bar: 5 cm. (**B**) Representative images of leaves treated with saline-alkaline stress for 14 days. Bar: 2 cm. (**C**) Leaf phenotype of the lower leaves under severe stress after 14 days of 200 mM saline-alkaline stress treatment. Bar: 2 cm. (**D**) Fresh weight of shoots. (**E**) Dry weight of shoots. (**F**) Fresh weight of roots. (**G**) Dry weight of roots. The x-axis indicates the treatment duration (7d and 14d) alongside the specific saline-alkaline concentrations, while the y-axis represents the biomass in grams (g). All data are expressed as the mean ± standard deviation (SD) of three independent biological replicates (*n* = 3). Different lowercase letters above the bars indicate statistically significant differences among the three treatment concentrations at the same time point, as determined by one-way ANOVA followed by Duncan’s multiple range test (*p* < 0.05).

*P. simonii* × *P. nigra* seedlings were treated with alkaline salt solutions of different concentrations for 7 and 14 days. Alkaline stress significantly inhibited growth (Figure 1A), with the severity of inhibition increasing with both concentration and duration. Leaf morphology was particularly affected (Figure 1B). Plants exposed to 200 mM for 14 days developed chlorosis and black spots on the lower portions of the leaves (Figure 1C). Interestingly, treatment with 50 mM saline-alkaline slightly promoted growth at 7 days but inhibited it by 14 days, whereas 200 mM consistently caused severe growth inhibition (Figure 1D–G). Although the mean fresh and dry weights of seedlings under the 50 mM treatment were slightly higher than those of the control, statistical analysis revealed no significant difference between them (*p* > 0.05).

**Figure 2:**
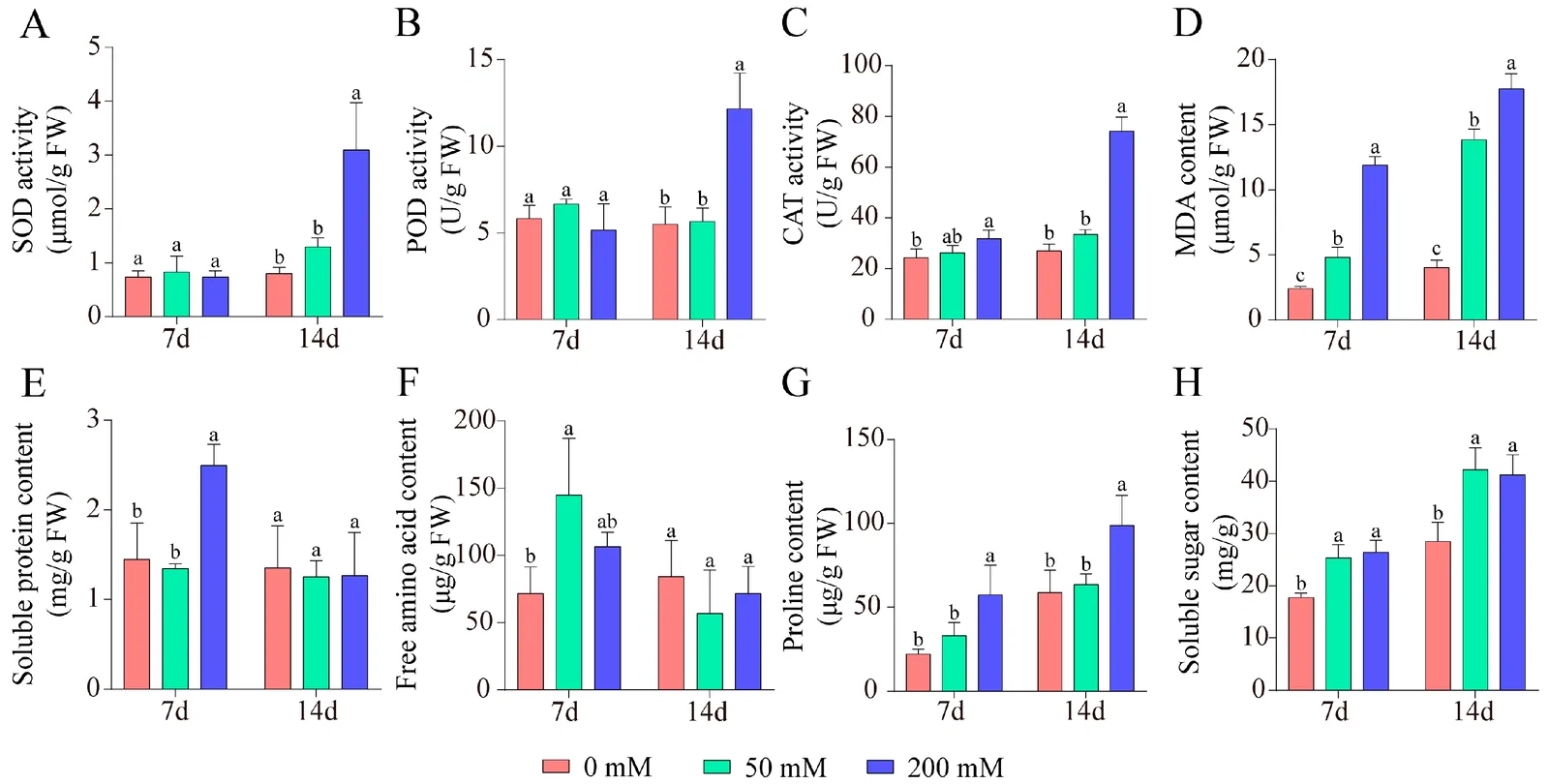
Effects of high- and low-concentration saline-alkaline stress on the physiology of *P. simonii* × *P. nigra*. (**A**–**C**) Activities of (**A**) superoxide dismutase (SOD), (**B**) peroxidase (POD), and (**C**) catalase (CAT) in the leaves. (**D**) Contents of malondialdehyde (MDA) in the leaves. (**E**–**H**) Contents of (**E**) soluble protein, (**F**) free amino acid, (**G**) proline, and (**H**) soluble sugar in the leaves. The x-axis indicates the treatment duration (7d and 14d) alongside the specific saline-alkaline concentrations (0, 50, and 200 mM). The y-axis represents the corresponding physiological measurements per gram of fresh weight (FW). All data are presented as the mean ± standard deviation (SD) of three independent biological replicates (*n* = 3). Different lowercase letters above the bars indicate statistically significant differences among the three treatment concentrations at the same time point, as determined by one-way ANOVA followed by Duncan’s multiple range test (*p* < 0.05).

Saline-alkaline stress also induced oxidative damage to the leaves of *P. simonii* × *P. nigra* (Figure 2). Activities of SOD, POD, and CAT increased significantly with increasing stress concentration and exposure duration, reaching their highest levels after 14 days of treatment with 200 mM saline-alkaline (Figure 2A–C). At the same time, MDA accumulated markedly (Figure 2D), suggesting that prolonged high-concentration stress caused oxidative damage despite the enhanced antioxidant defense response despite enhanced antioxidant activity. Analysis of osmoregulatory substances revealed that soluble protein, free amino acids, proline, and soluble sugars all increased significantly with both concentration and duration of stress (Figure 2E–H). These findings indicate that *P. simonii* × *P. nigra* actively accumulates multiple osmoregulatory substances to maintain cellular osmotic balance and enhance tolerance under saline-alkaline stress.

### 3.2. Transcriptome Analysis of P. simonii × P. nigra Leaves Under High and Low Saline-Alkaline Stress

**Figure 3:**
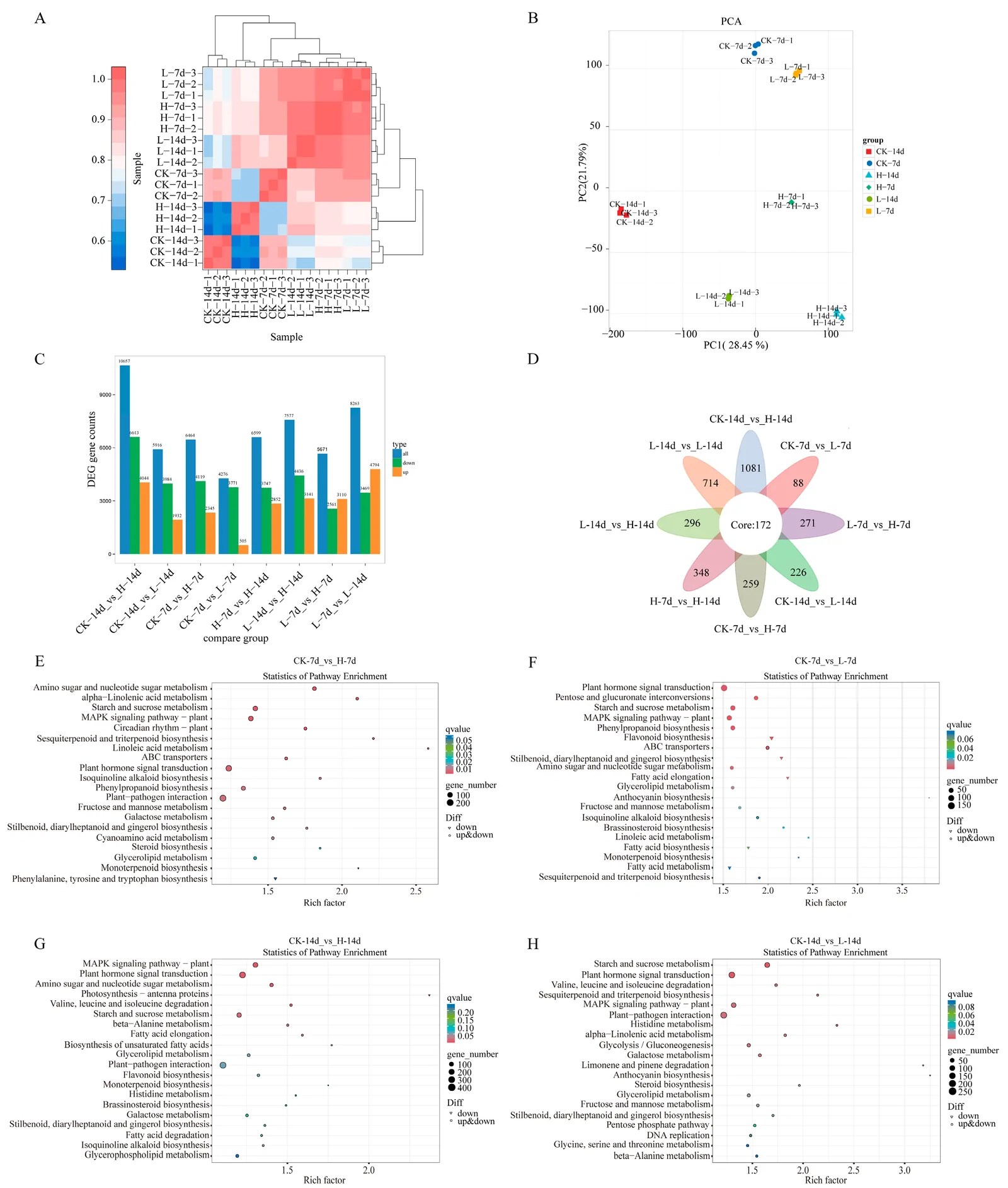
RNA-seq analysis of *P. simonii* × *P. nigra* leaves under high and low saline-alkaline stress. (**A**) Correlation analysis of six groups of samples. The color scale indicates the correlation coefficient, with darker red representing higher reproducibility between replicates. (**B**) Principal component analysis (PCA) plot demonstrating the global expression variations among different samples. (**C**) The number of differentially expressed genes (DEGs) in eight comparison groups was subjected to statistical analysis. The y-axis shows the gene counts, with orange and green bars representing upregulated and downregulated DEGs, respectively. (**D**) Venn diagram illustrating the unique and overlapping DEGs among the core comparison groups. (**E**) KEGG analysis of DEGs in CK-7d vs. H-7d. (**F**) KEGG analysis of DEGs in CK-7d vs. L-7d. (**G**) KEGG analysis of DEGs in CK-14d vs. H-14d. (**H**) KEGG analysis of DEGs in CK-14d vs. L-14d. The y-axis represents the pathway names, and the x-axis represents the rich factor. Bubble size indicates the number of DEGs in a specific pathway, and the color gradient represents the significance level.

RNA sequencing was conducted on leaves of *P. simonii* × *P. nigra* subjected to six treatments: 7-day control (CK-7d), 7-day high alkaline (H-7d), 7-day low alkaline (L-7d), 14-day control (CK-14d), 14-day high alkaline (H-14d), and 14-day low alkaline (L-14d). In total, 111.84 Gb of clean data were obtained. The sequencing depth was robust, generating between 19.42 million and 23.99 million clean pair-end reads per sample, corresponding to ≥5.80 Gb per sample. Sequencing quality was exceptionally high, with Q30 values ranging from 97.57% to 99.37%. Furthermore, the clean reads were aligned to the *Populus trichocarpa* reference genome, achieving high mapping efficiency. Total mapped reads accounted for 84.78% to 89.41% of the clean reads across all samples, and uniquely mapped reads ranged from 83.37% to 87.70%. These metrics ensure high reliability for downstream analyses. Biological replicates showed strong consistency (correlation coefficient ≥ 0.8) (Figure 3A). Principal component analysis (PCA) revealed that PC1 and PC2 explained 28.45% and 21.79% of the total variance, respectively, clearly distinguishing the six sample groups (Figure 3B). Differential expression analysis (fold change (FC) ≥ 2 and FDR < 0.01) identified large sets of DEGs across comparisons: 6464 in CK-7d vs. H-7d (2345 upregulated, 4119 downregulated), 4276 in CK-7d vs. L-7d (505 upregulated, 3771 downregulated), 10,657 in CK-14d vs. H-14d (4044 upregulated, 6613 downregulated), 5916 in CK-14d vs. L-14d (1932 upregulated, 3984 downregulated), 5671 in L-7d vs. H-7d (3110 upregulated, 2561 downregulated), 7577 in L-14d vs. H-14d (3141 upregulated, 4436 downregulated), 6599 in H-7d vs. H-14d (2852 upregulated, 3747 downregulated), and 8263 in L-7d vs. L-14d (4794 upregulated, 3469 downregulated) (Figure 3C). Venn diagram analysis revealed 172 DEGs shared across all eight comparisons (Figure 3D). These results demonstrate that both concentration and duration of saline-alkaline stress induce extensive, distinct changes in gene expression in *P. simonii* × *P. nigra*. To identify metabolic pathways associated with DEGs in *P. simonii* × *P. nigra* leaves under saline-alkaline stress, KEGG enrichment analysis was performed for four comparisons: CK-7d vs. H-7d, CK-7d vs. L-7d, CK-14d vs. H-14d, and CK-14d vs. L-14d. DEGs were mainly enriched in pathways related to amino sugar and nucleotide sugar metabolism, alpha-linolenic acid metabolism, valine/leucine/isoleucine degradation, starch and sucrose metabolism, MAPK signaling pathway, plant hormone signal transduction, phenylpropanoid biosynthesis, photosynthesis-antenna proteins, pentose and glucuronate interconversions, and sesquiterpenoid and triterpenoid biosynthesis (Figure 3E–H).

### 3.3. Metabolome Analysis of P. simonii × P. nigra Leaves Under High and Low Saline-Alkaline Stress

**Figure 4:**
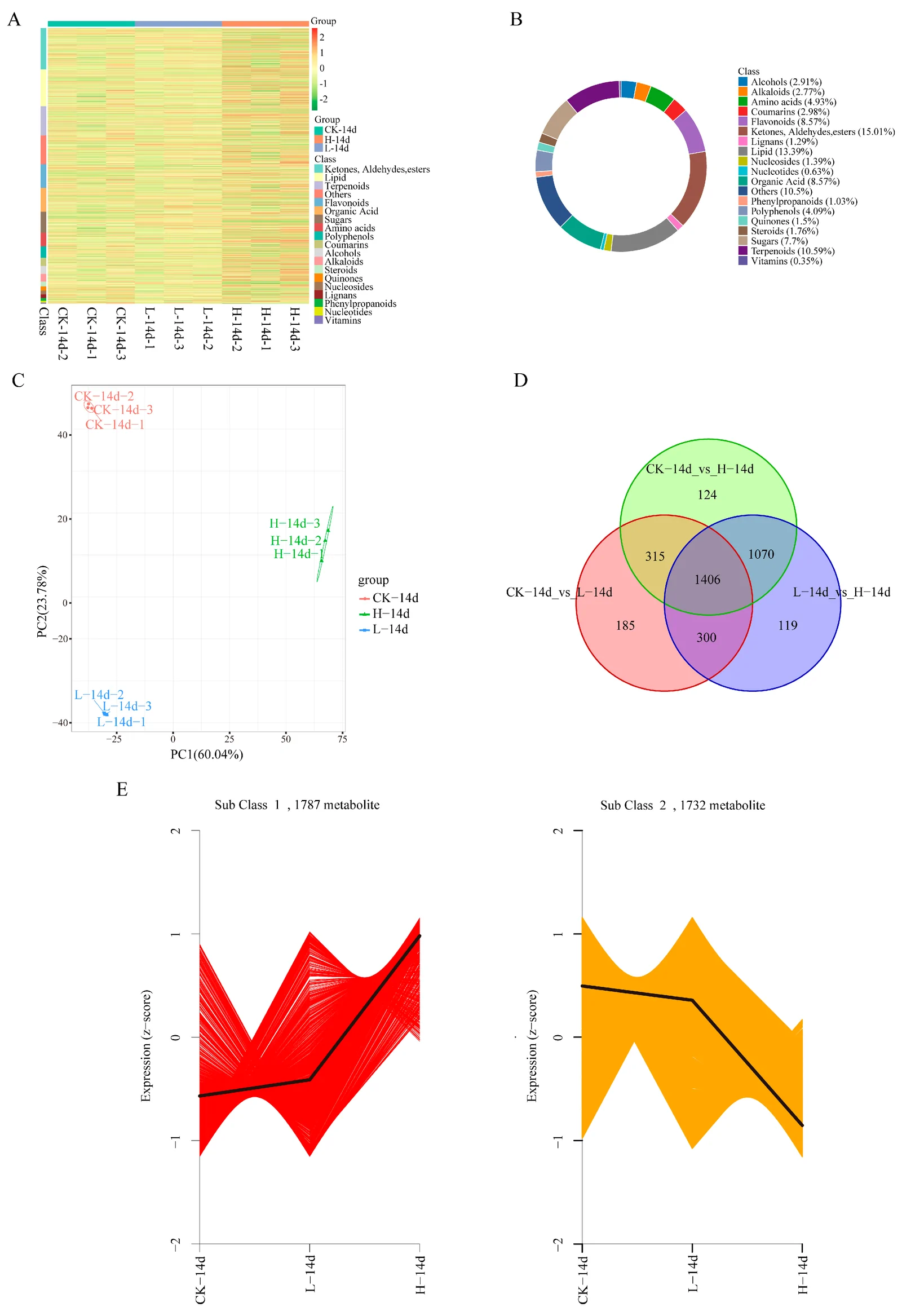
Metabolomics analysis of *P. simonii* × *P. nigra* leaves under high and low saline-alkaline stress. (**A**) Hierarchical clustering heatmap of all identified metabolites across different samples. The color scale represents the normalized relative abundance of metabolites, with red indicating higher accumulation and blue indicating lower accumulation. (**B**) Categories and proportions of different metabolics. (**C**) PCA analysis of different samples. (**D**) Venn diagram of differential accumulated metabolites (DAMs) between CK-14d, L-14d and H-14d. (**E**) The K-means cluster analysis on DAMs. The x-axis represents different concentrations of saline-alkaline treatment groups: CK-14d (control, 0 mM), H-14d (high saline-alkaline, 200 mM), and L-14d (low saline-alkaline, 50 mM), the y-axis represents the standardized expression level (Z-score) of metabolites. Different colors represent different clusters, and DAMs in the same cluster have similar expression change patterns.

Transcriptomic analysis showed that 14-day saline-alkaline stress induced the most pronounced changes in DEG number and expression. Therefore, metabolomic profiling was performed on leaves from CK-14d (control, 0 mM), H-14d (high saline-alkaline, 200 mM), and L-14d (low saline-alkaline, 50 mM) leaves. Across nine samples, 4257 metabolites were detected (Figure 4A) and grouped into 19 categories. The major classes included ketones (15.01%), aldehydes (13.39%), esters (10.59%), lipids (8.57%), terpenoids (8.57%), flavonoids (7.7%), organic acids (4.93%), sugars (4.09%), amino acids (2.98%), polyphenols (2.91%), coumarins (2.77%), alcohols (2.91%), and alkaloids (2.77%) (Figure 4B). PCA showed clear separation among treatments, with PC1 and PC2 explaining 60.04% and 23.78% of the variance, respectively, confirming distinct metabolite profiles under high and low saline-alkaline stress (Figure 4C). OPLS-DA further identified 2915, 2206, and 2895 differential accumulated metabolites (DAMs) in CK-14d vs. H-14d, CK-14d vs. L-14d, and L-14d vs. H-14d, respectively, with 1406 DAMs shared across comparisons (Figure 4D). K-means clustering revealed two major accumulation patterns, containing 1787 (subclass 1) and 1732 (subclass 2) metabolites, respectively (Figure 4E).

### 3.4. Quantitative Analysis of DAMs and KEGG Enrichment Analysis

**Figure 5:**
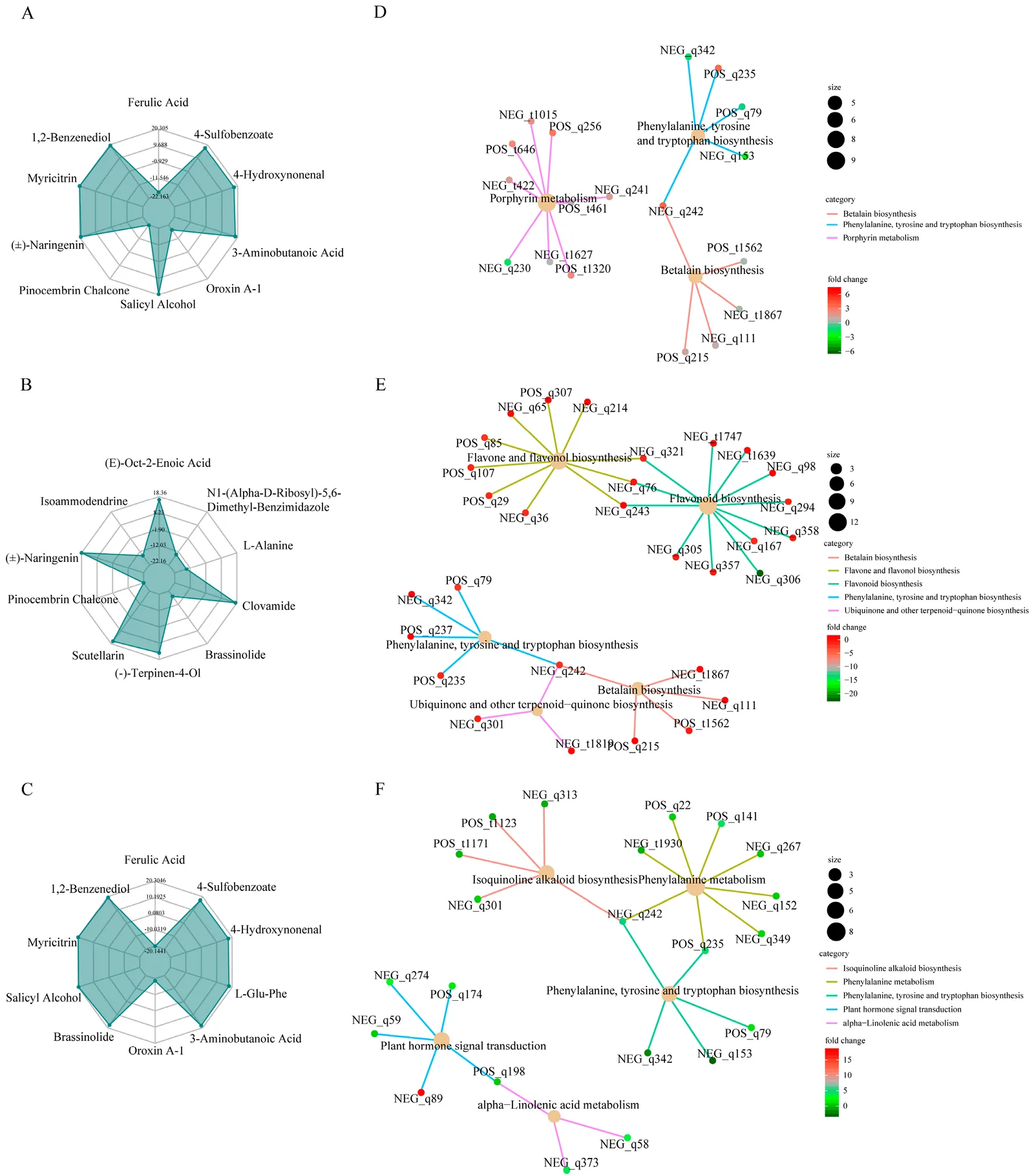
Quantitative and KEGG analysis of DAMs. (**A**) Radar chart of DAMs for CK-14d vs. H-14d. (**B**) Radar chart of DAMs for CK-14d vs. L-14d. (**C**) Radar chart of DAMs for L-14d vs. H-14d. Each axis of the radar chart represents different categories of DAMs, and the axis values represent the relative accumulation of DAMs in that category. (**D**) KEGG analysis of DAMs in CK-14d vs. H-14d. (**E**) KEGG analysis of DAMs in CK-14d vs. L-14d. (**F**) KEGG analysis of DAMs in L-14d vs. H-14d. The light yellow nodes in the figure represent pathways, and the small nodes connected to them are the specific metabolites annotated to the corresponding pathways. The size of the nodes indicates the number of differential metabolites annotated to the pathway, and the depth of the color indicates the fold change.

To further clarify the metabolic pathways associated with DEGs under saline-alkaline stress, quantitative and KEGG enrichment analyses were performed on DAMs. Compared with controls, high-concentration saline-alkaline stress significantly increased the accumulation of amino acids, organic acids, sugars, alkaloids, flavonoids, and terpenoids, while reducing lipids, ketones, aldehydes, and esters (Table S2). According to the transcriptome results, it was found that exposure to saline-alkaline stress in *P. simonii* × *P. nigra* significantly caused changes in the transcription levels of genes involved in starch and sucrose metabolism, flavonoid biosynthesis, and amino acid metabolism pathways. Therefore, we analyzed the changes in the content of carbohydrates, flavonoids, and amino acid metabolites, and the results are shown in Table S2. Under high-concentration saline-alkaline stress, the contents of D-gluconic acid, D-arabinose, perseitol, raffinose, D-fructose, myricitrin, rhamnetin, (±)-naringenin, (−)-catechin, (±)-catechin hydrate, and isoquercitrin in the leaves of *P. simonii* × *P. nigra* were significantly increased and the contents of D-glucose 6-phosphate, D-fructose-6-phosphate (disodium) salt, b-D-glucopyranoside, (3b,5a,25S)-spirostan-3-yl 6-O-b-D-galactopyranosyl-(9CI), xylotriose, (2R,3S,4S,5S,6R)-6-(hydroxymethyl) oxane-2,3,4,5-tetrol, pinocembrin chalcone, albanin A, camelliquercetiside B, apigenin-4′-O-rhamnoside, and 2′-methoxyflavone were significantly decreased. Amino acid compounds such as Dl-tryptophan, D-leucine, L-leucine, D-proline, L-tyrosine, L-proline, and Dl-3-phenylalanine showed strong increases under high-concentration saline-alkaline stress, the content of D-ornithine (hydrochloride), glutamyl-S-methylcysteine, L-alanyl-L-alanyl-L-alanine, L-glutamic acid, N-((-)-jasmonoyl)-S-isoleucine and His-Pro were significantly decreased. The top 10 DAMs by |log_2_FC| were visualized in radar charts. After 14 days at 200 mM, metabolites including 1,2-benzenediol, myricitrin, (±)-naringenin, salicyl alcohol, 3-aminobutanoic acid, 4-hydroxynonenal, and 4-sulfobenzoate were significantly upregulated, while ferulic acid, pinocembrin chalcone, and oroxin A-1 were downregulated (Figure 5A). Under 50 mM stress, (E)-oct-2-enoic acid, (±)-naringenin, scutellarin, (−)-terpinen-4-ol, and clovamide accumulated, whereas isoammoderine, pinocembrin chalcone, brassinolide sterols, L-alanine, and N1-(alpha-D-ribosyl)-5,6-dimethyl-benzimidazole declined (Figure 5B). Compared with low stress, high concentration treatment caused marked accumulation of 1,2-benzenediol, myricirin, salicyl alcohol, brassinolide, 3-aminobutanoic acid, γ-Glu-Phe, 4-hydroxynonenal, and 4-sulfobenzoate, while ferulic acid and oroxin A-1 were reduced (Figure 5C). KEGG enrichment of DAMs revealed that CK-14d vs. H-14d was enriched in betalain biosynthesis, porphyrin metabolism, and phenylalanine/tyrosine/tryptophan biosynthesis (Figure 5D). CK-14d vs. L-14d was enriched in betalain biosynthesis, flavone and flavonol biosynthesis, flavonoid biosynthesis, phenylalanine/tyrosine/tryptophan biosynthesis, and ubiquinone and other terpenoid quinone biosynthesis (Figure 5E). L-14d vs. H-14d was enriched in isoquinoline alkaloid biosynthesis, phenylalanine metabolism, phenylalanine/tyrosine/tryptophan biosynthesis, plant hormone signal transduction, and alpha linolenic acid metabolism (Figure 5F). Across all comparisons, phenylalanine, tyrosine, and trypsin biosynthesis emerged as a consistently enriched pathway.

### 3.5. Joint Analysis of DEGs and DAMs

**Figure 6:**
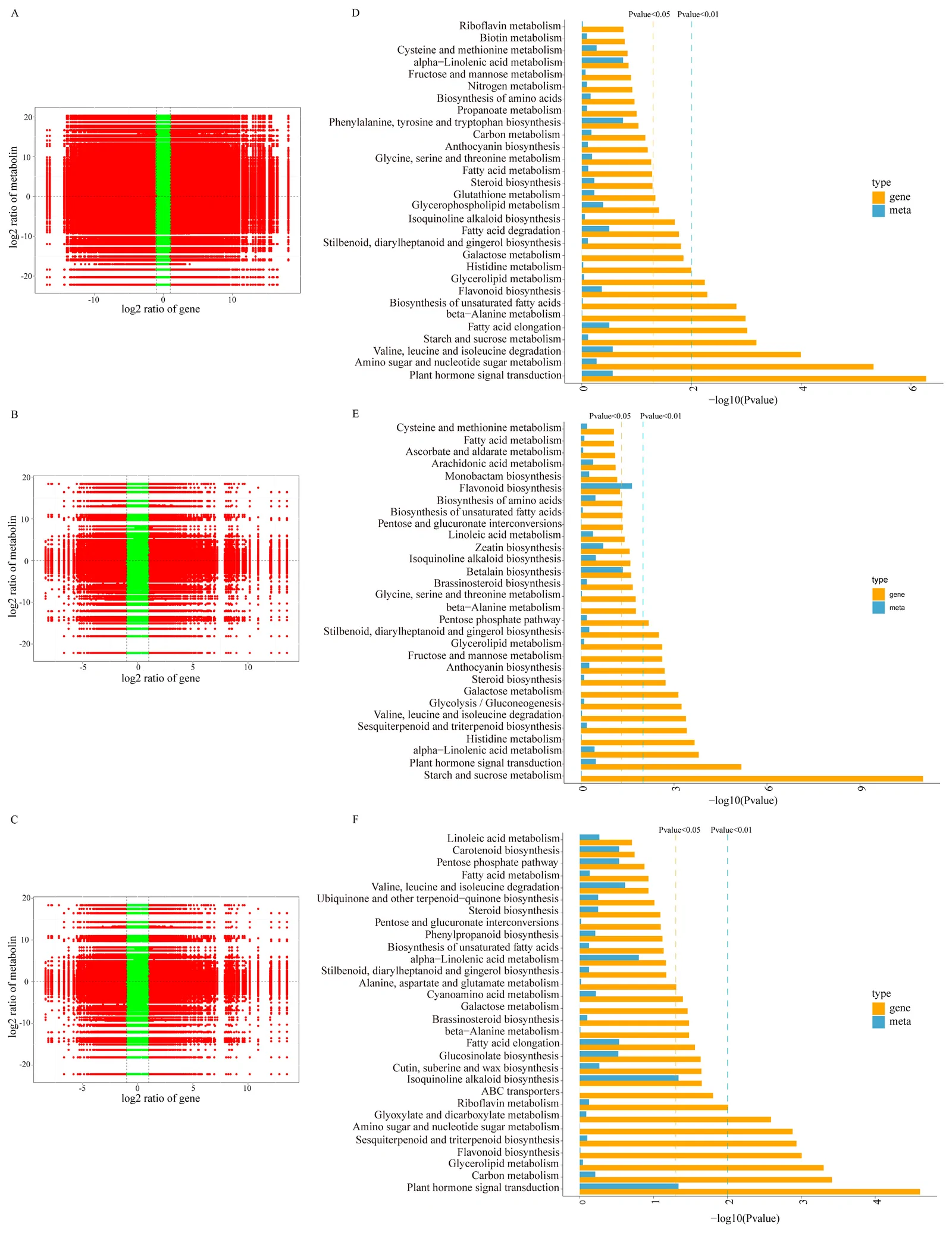
Correlation analysis between transcriptome and metabolome. (**A**–**C**) Nine-quadrant plots illustrating the global association between differentially expressed genes (DEGs) and differentially accumulated metabolites (DAMs) in the comparisons of (**A**) CK-14d vs. H-14d, (**B**) CK-14d vs. L-14d and (**C**) L-14d vs. H-14d. The x-axis represents the log_2_ (fold change) of transcripts, and the y-axis represents the log_2_ (fold change) of metabolites. The order of quadrants 1 to 9 is from left to right and top to bottom. Data points in quadrants 3 and 7 represent genes and metabolites with consistent regulatory trends; quadrants 1 and 9 represent opposite expression trends of genes and metabolites; quadrants 2 and 8 represent no change in genes but upregulation or downregulation of metabolites; quadrants 4 and 6 represent no change in metabolites but upregulation or downregulation of genes; quadrant 5 represents no significant changes in either metabolites or genes. (**D**–**F**) KEGG enrichment analysis of DEG and DEM in (**D**) CK-14d vs. H-14d, (**E**) CK-14d vs. L-14d and (**F**) L-14d vs. H-14d. Each bar in the figure represents a KEGG pathway, and different colors indicate different omics, yellow represents the transcriptome, and blue represents the metabolome. The x-axis indicates the significance of pathway enrichment, the y-axis indicates the name of the pathway, the higher the bar, the more significantly the biological pathway is enriched in the tested samples.

To examine the relationship between transcriptional and metabolic changes in *P. simonii* × *P. nigra* leaves under saline-alkaline stress, a correlation analysis was performed between gene expression and metabolite levels. No significant differences were observed in the 5th quadrant. In quadrants 3 and 7, gene and metabolite trends were consistent, suggesting positive regulation, whereas in quadrants 1 and 9, trends were opposite, indicating negative correlations. In CK-14d vs. H-14d, CK-14d vs. L-14d, and L-14d vs. H-14d, the number of genes and metabolites located in quadrant 1 were 1951/952, 6353/1153, and 2553/1356; in quadrant 3 these were 1468/1456, 4999/732, and 4873/1276; in quadrant 7 they were 4991/492, 3352/1606, and 2572/1282; and in quadrant 9 they were 1982/1355, 1953/1640, and 1978/1324 (Figure 6A–C). Compared with high-concentration stress, low-concentration stress placed more genes and metabolites in quadrants 1 and 3, suggesting stronger coordinated transcriptional-metabolic changes (Figure 6A,B). KEGG enrichment analysis revealed that under high-concentration stress, DEGs were enriched in plant hormone signal transduction, flavonoid biosynthesis, and carbon metabolism, while DAMs were significantly enriched in isoquinoline alkaline biosynthesis and plant hormone signal transduction (Figure 6D–F). Under low-concentration stress, DEGs were enriched in starch and sucrose metabolism and plant hormone signal transduction, whereas DAMs were significantly enriched in flavonoid and betalain biosynthesis (Figure 6E).

### 3.6. Correlation Analysis of DEGs and DAMs

**Figure 7:**
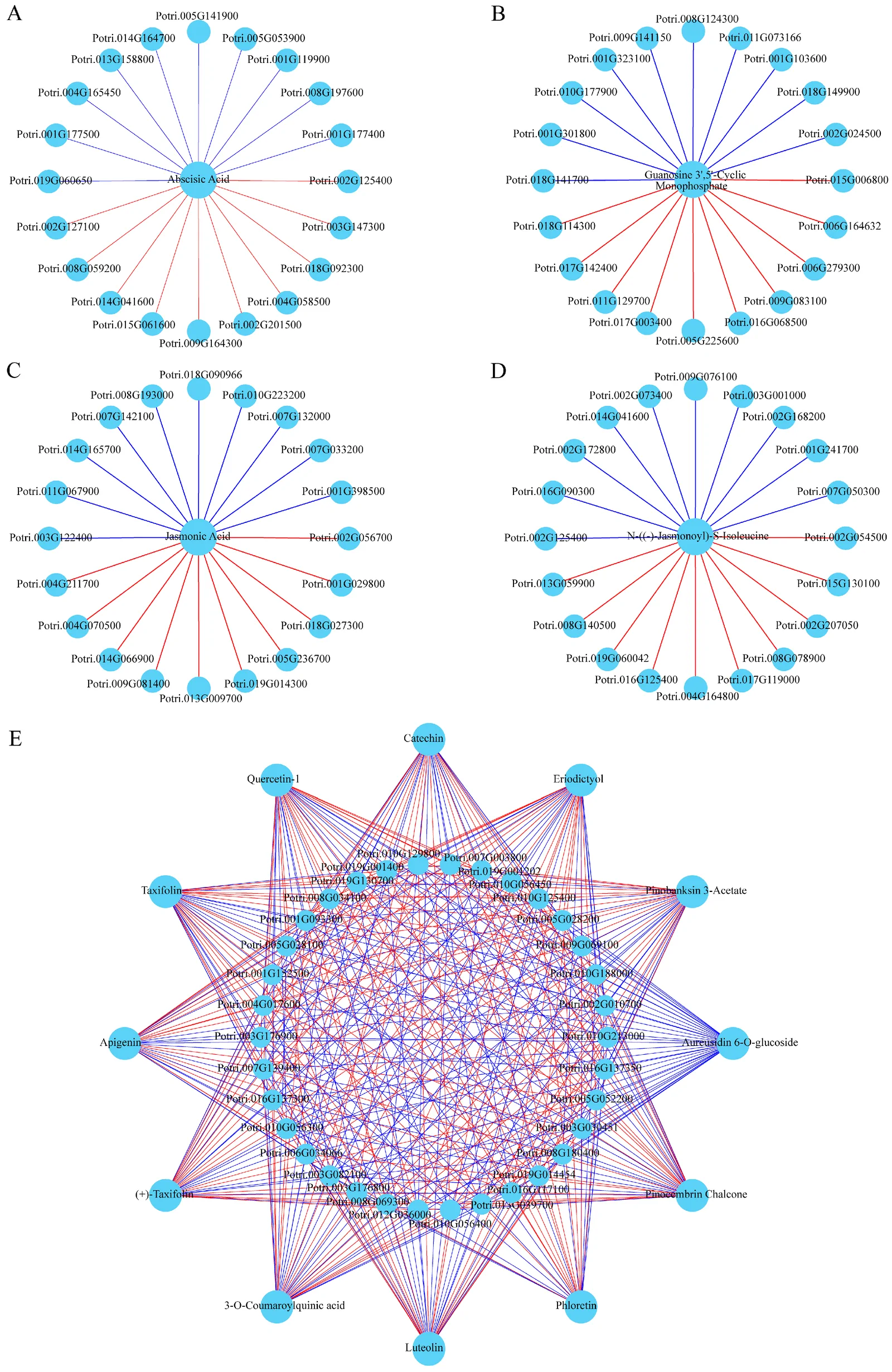
Correlation analysis of DEGs and DAMs. (**A**–**D**) Correlation networks between specific DEGs and endogenous stress hormones enriched in the plant hormone signal transduction pathway. (**E**) Correlation network between specific structural DEGs and DAMs enriched in the flavonoid biosynthesis pathway. The nodes represent individual genes or metabolites, and the connecting lines (edges) indicate significant regulatory relationships. Red lines represent positive correlations, while blue lines represent negative correlations.

We conducted correlation network analysis on DEGs and DAMs significantly enriched in plant hormone signal transduction and flavonoid biosynthesis pathways in the CK-14d vs. H-14d and CK-14d vs. L-14d control groups, respectively. Among them, 1568 genes and 4 metabolites are mainly involved in the biosynthesis of abscisic acid, jasmonic acid, N-((-)-jasmonoyl)-S-isoleucine and guanosine 3′,5′-cyclic monophosphate in the plant hormone signaling pathway (Figure 7A–D). The transcription levels of Potri.002G125400(ABF2), Potri.003G147300(MYC4), Potri.018G092300(IAA33), Potri.004G058500(CAMRLK), Potri.002G201500(ERS1), Potri.009G164300(TGA9), Potri.014G041600(BZR2), Potri.008G059200(PP2CA) and Potri.002G127100(BRI1) were significantly positively correlated with abscisic acid accumulation, the transcription level of Potri.019G060650(MIK2), Potri.001G177500(IAA19), Potri.004G165450(AXX17), Potri.014G164700(AHK2), Potri.005G141900(CYCD3-3), Potri.005G053900(IAA30), Potri.008G197600(AHP1) and Potri.001G177400(IAA16) were significantly negatively correlated with abscisic acid accumulation (Figure 7A). After 14 days of high concentration alkali stress, the content of jasmonic acid in the leaves of *P. simonii* × *P. nigra* was positively correlated with the transcription levels of Potri.002G056700(MYB), Potri.001G029800(TGA2), Potri.018G027300(SD25), Potri.005G236700(ARF5), Potri.019G014300(EIN4), Potri.013G009700(CKI1), Potri.009G081400(bHLH25), Potri.014G066900(SAUR59), Potri.004G070500(ESD4), and Potri.004G211700(ARF10). However, the transcription levels of Potri.003G122400(SCL28), Potri.011G067900(CAMRLK), Potri.014G165700(SLY2), Potri.007G142100(integrin-linked protein kinase), Potri.008G193000(ARR4), Potri.018G090966(PR5K), Potri.010G223200(ARF16), Potri.007G132000(SGR7) and Potri.001G398500(PSKR2) were negatively correlated with jasmonic acid accumulation (Figure 7C). As shown in Figure 7E, 34 genes and 12 metabolites were mainly involved in the flavonoid biosynthesis pathway. Among them, the transcription levels of Potri.010G213000(CHI1), Potri.005G052200(CFI1), Potri.005G028200(AXX17), and Potri.010G125400(TKPR2) were significantly positively correlated with the contents of aureusidin 6-O-glucoside, pinocembrin chalcone and eriodictyol, while the transcription levels of Potri.010G12980(KMD1), and Potri.001G093300(TT2) were significantly negatively correlated with the accumulation of pinocembrin chalcone and eriodictyol.

To move beyond descriptive pathway co-enrichment and identify concrete mechanistic linkages between transcription and metabolism under saline-alkaline stress, we focused on highly correlated gene–metabolite pairs with clear biological relevance within the plant hormone signal transduction and flavonoid biosynthesis networks (Figure 7). In the flavonoid biosynthesis pathway (Figure 7E), we identified a direct functional linkage between specific structural genes and antioxidant metabolite accumulation. A strong positive correlation was observed between the transcriptional activation of key enzymes, specifically Potri.010G213000(CHI1), Potri.005G052200(CFI1), and Potri.010G125400(TKPR2), and the significant accumulation of downstream flavonoids, including pinocembrin chalcone, eriodictyol, and aureusidin 6-O-glucoside. Conversely, the transcription of negative regulators such as Potri.001G093300(TT2) and Potri.010G12980(KMD1) was significantly negatively correlated with the accumulation of these metabolites. This specific pairing provides a clear mechanistic explanation: the downregulation of repressors like TT2 coupled with the specific activation of CHI1 drives metabolic flux toward flavonoid accumulation, thereby functioning as a crucial ROS scavenging mechanism to mitigate saline-alkaline stress.

Furthermore, a highly coordinated feedback mechanism was observed between hormone accumulation and signal transduction (Figure 7A–D). The robust accumulation of ABA was strongly positively correlated with the transcriptional activation of its core signaling and response components, most notably Potri.002G125400(ABF2), Potri.008G059200(PP2CA), and Potri.014G041600(BZR2). Similarly, under severe high-concentration stress at 14 days, the accumulation of JA perfectly mirrored the substantial upregulation of key defense-related transcription factors and signaling genes, including Potri.003G147300(MYC4), Potri.002G056700(MPH1), and Potri.019G014300(EIN4). These tight, biologically relevant correlations illustrate a clear mechanistic linkage where the metabolic accumulation of stress hormones (ABA and JA) directly dictates the transcriptional reprogramming of downstream signaling cascades to mount a coordinated osmotic and defensive response.

### 3.7. WGCNA Analysis

**Figure 8:**
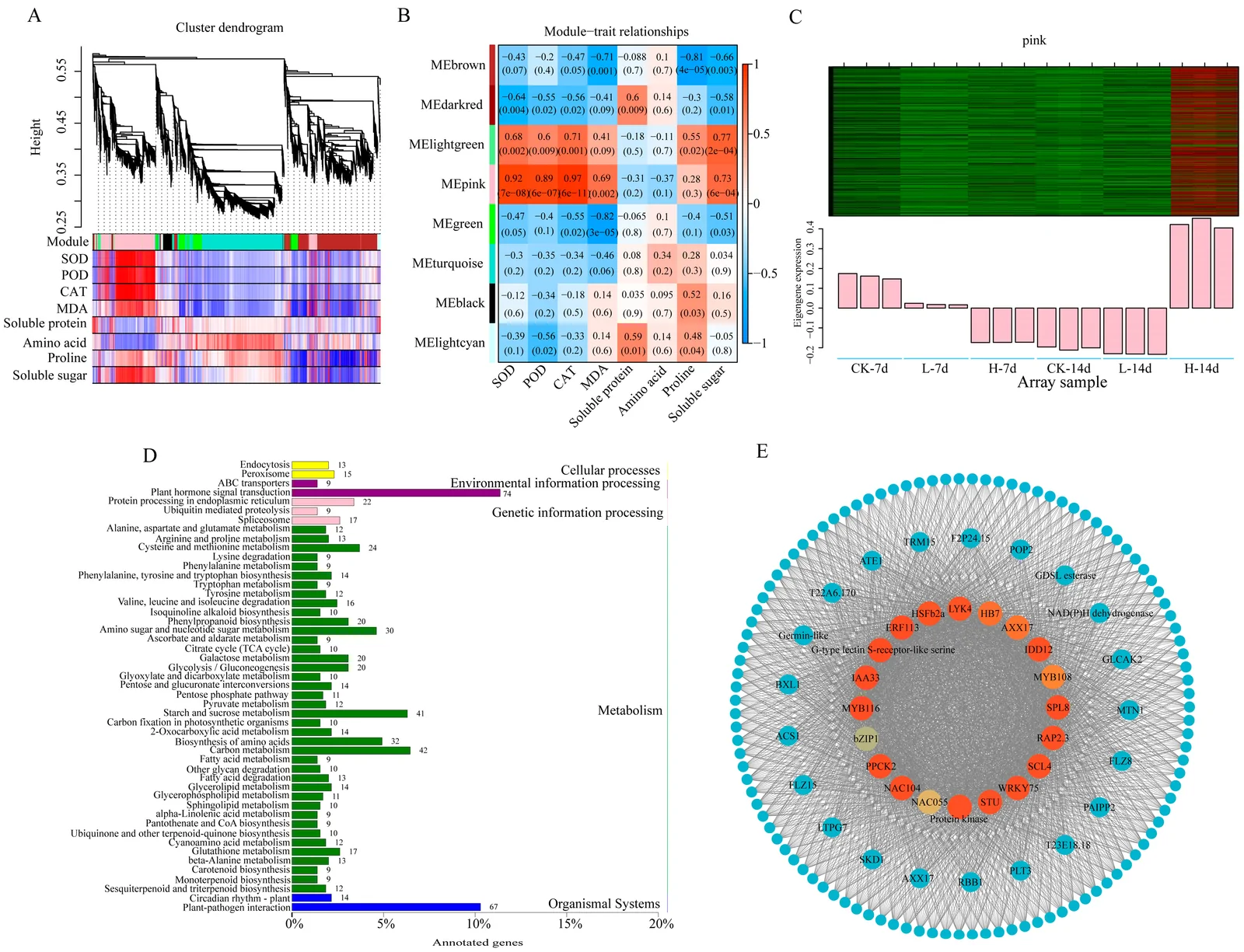
Weighted gene co-expression network analysis (WGCNA) of differentially expressed genes (DEGs) and physiological traits in *P. simonii* × *P. nigra* leaves under varying intensities of saline-alkaline stress. (**A**) Hierarchical clustering tree of co-expression modules based on WGCNA for DEGs. Branches correspond to eight distinct co-expression modules, designated by different colors. (**B**) Heatmap of gene module–physiological index correlations. Each row represents a specific gene module, and each column represents a physiological index. The color gradient indicates the Pearson correlation coefficient (r), with red representing positive correlations and blue representing negative correlations. (**C**) Expression patterns of DEGs in the “pink” module. red indicates up-regulated differentially expressed genes, and green indicates down-regulated DEGs. (**D**) KEGG analysis of DEGs in the “pink” module. The y-axis shows the enriched pathways, and the x-axis represents the enrichment ratio. (**E**) Co-expression network of hub-genes in the “pink” module. The nodes represent individual genes, and the connecting lines (edges) indicate strong co-expression relationships.

To uncover the gene regulatory network of *P. simonii* × *P. nigra* under saline-alkaline stress, 17,093 DEGs were analyzed together with physiological indices including SOD, POD, and CAT activities and the contents of MDA, soluble sugars, soluble proteins, proline, and free amino acids using WGCNA. Hierarchical clustering divided the genes into eight co-expression modules with distinct expression patterns (Figure 8A). Among these, the “brown”, “lightgreen”, and “pink” modules contained 210,578, and 1631 genes, respectively. The “lightgreen” and “pink” modules were positively correlated with antioxidant enzyme activity and soluble sugar content, whereas the “brown” module was negatively correlated with MDA and proline (Figure 8B). Because the “pink” module exhibited the most robust stress-responsive upregulation under prolonged high-concentration stress and was highly enriched in core regulatory genes, it was selected as the primary focus for our hub gene analysis (Figure 8C,D). Expression patterns showed that “pink” module genes were downregulated after 7 days of stress and after 14 days of low-concentration treatment, but strongly upregulated after 14 days of high-concentration stress. The expression dynamics and functional enrichment of the auxiliary “brown” and “lightgreen” modules are detailed in Figure S1. Gene co-expression analysis showed that multiple transcription factor families, including HSFb2a, ERF113, MYB116, bZIP1, NAC104, NAC055,WRKY75, RAP2.3, SPL8, MYB108, and HB7, were significantly enriched in the hub genes of the pink module (Figure 8E). We selected nine hub genes to perform RT-qPCR. As shown in Figure S2, PsnHB7, PsnMYB108, PsnNAC055, PsnPPCK2, and PsnSCL4 were significantly upregulated under saline-alkaline stress. Notably, under severe stress conditions, the relative expression levels of these hub genes exhibited a marked increase with prolonged stress duration and intensity. The expression patterns of these genes were consistent with the RNA-seq data, underscoring their critical roles in the saline-alkaline stress response in *P. simonii* × *P. nigra*. These findings suggest that saline-alkaline stress may facilitate plant adaptation by modulating key transcription factors involved in hormone signaling pathways.

### 3.8. Effects on Genes Involved in Primary and Secondary Metabolism

**Figure 9:**
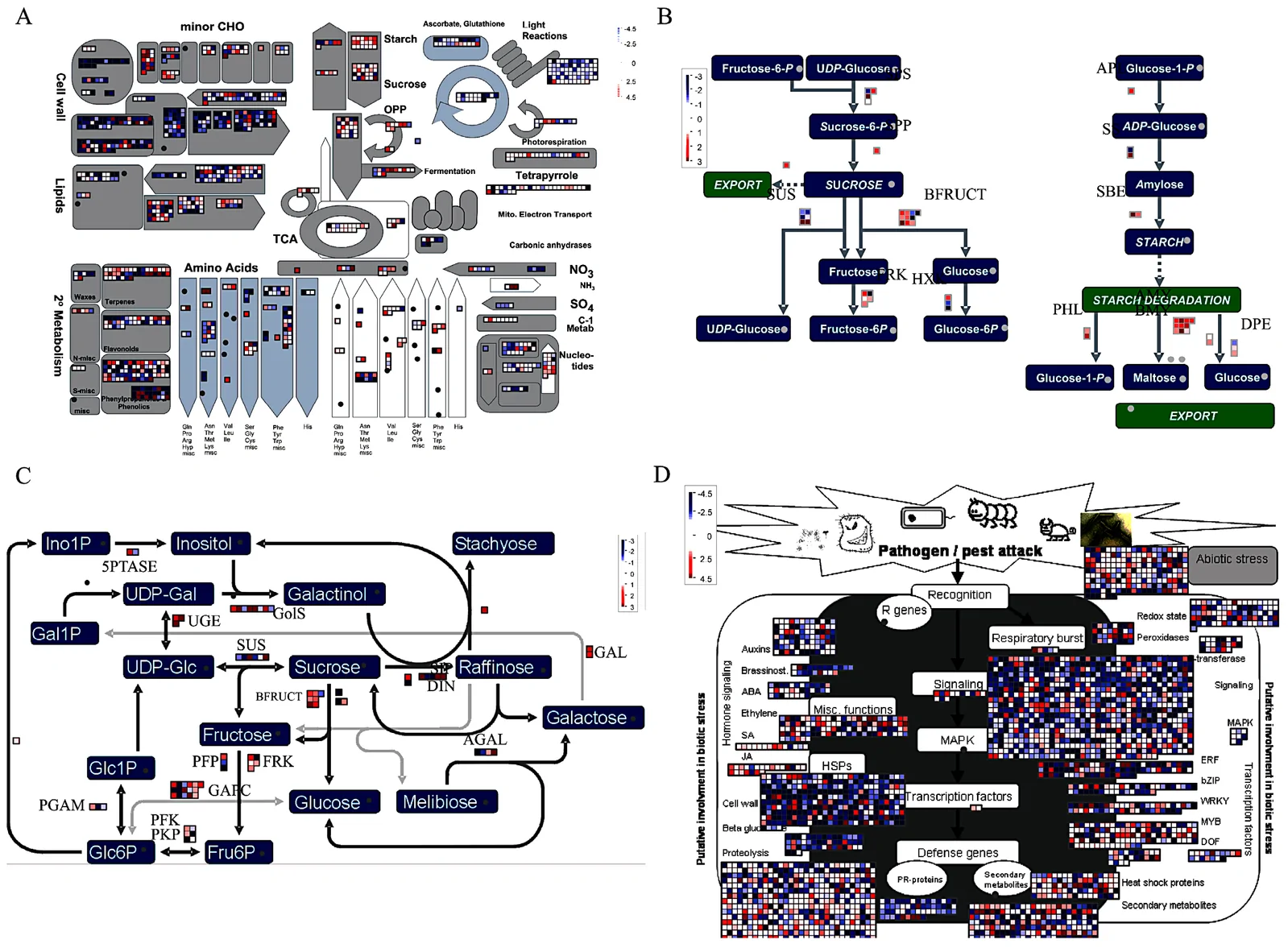
Pathway mapping of DEGs. (**A**) Overview pathway mapping of DEGs in CK-14d vs. H-14d. (**B**) Starch and sucrose degradation pathway mapping of DEGs in CK-14d vs. H-14d. (**C**) Raffinose metabolism pathway mapping of DEGs in CK-14d vs. H-14d. (**D**) Abiotic stress mapping of DEGs in CK-14d vs. H-14d. Heatmaps for individual genes were based on log2 (fold change) values, where red indicates upregulated expression and blue represents downregulated expression.

Under low-concentration saline-alkaline stress, genes associated with light reactions, sucrose/starch and carbohydrate metabolism, amino acid synthesis and metabolism, and raffinose metabolism were upregulated in *P. simonii* × *P. nigra* leaves, whereas genes related to cell wall biomass synthesis and degradation, secondary metabolism, and lipid metabolism were downregulated (Figure S3A). Under high-concentration stress, more DEGs were detected in sucrose/starch degradation, raffinose metabolism, amino acid biosynthesis and degradation, and secondary metabolism, with changes more pronounced than under low-concentration stress. In contrast, genes linked to light reactions, cell wall synthesis/degradation, and phenylpropanoid metabolism were significantly downregulated (Figure 9A). Otherwise, genes related to starch and sucrose degradation were significantly upregulated in *P. simonii* × *P. nigra* leaves under low-concentration saline-alkaline stress, including Potri.018g025100(SPS1F), Potri.002g014300(AMY2), Potri.017g040800(BAM5), Potri.009g118800(APL3), Potri.005g251000(SBE2.2) and Potri.002g202300(SUS3). However, Potri.018g063500(SUS4) and Potri.015g127100(BFRUCT3) in the leaves showed downregulation (Figure S3B, Table S3). Under high-concentration stress, the transcription level of Potri.018g025100(SPS1F), Potri.006g064300(SPS3F), Potri.005g251000(SBE2.2), Potri.008g101500(BFRUCT), Potri.004g081300(SUS6), Potri.002g014300(AMY2), Potri.008g174100(BAM1), Potri.008g204200(BAM9) and Potri.001g148900(BAM3) involving starch and sucrose degradation were significantly increased, while the transcription level of Potri.015g127100(BFRUCT3), Potri.005g238600(HXK3) and Potri.017g139100(SUS5) were significantly decreased (Figure 9B, Table S3). Genes involved in raffinose metabolism were significantly upregulated under low-concentration saline-alkaline stress, such as Potri.010g150400, Potri.008g101000(GolS1), Potri.018g126400(DIN10) and Potri.010g118200 (AGAL2) (Figure S3C, Table S4). Under high-concentration stress, the transcription level of Potri.008g101000(GS2), Potri.010g150400(GolS1), Potri.005g006800(GolS2), Potri.006g052800(SIP2), Potri.006g065700(RFS6), Potri.018g126400(DIN10), Potri.001g090700(UGE5), and Potri.008g179300(GAPC1) involved in raffinose metabolism were significantly increased, while the transcription level of Potri.004g038100(AGAL2), Potri.011g015900(PFP) and Potri.001g289300(PPK1), Potri.009g084700(PPK3) were significantly decreased (Figure 9C, Table S4).

Both high and low concentrations of saline-alkaline stress induced transcriptional changes in abiotic stress-related genes, including, Potri.013g154400(PER1), Potri.013g083600(PER7), Potri.012g082800(GrxC), Potri.010g061100(GSTU1), Potri.001g416500(TRX-H), Potri.011g140600(GSTU22), Potri.017g138800(GSTF12) and, Potri.011g140700(GSTU19) related to glutathione S-transferase, peroxidase, and redox responses (Figure 9D and Figure S3C, Table S5). In hormone signal transduction, saline-alkaline stress significantly upregulated the expression of Potri.014g028200(ABF2), Potri.009g101200(ABF3), Potri.002g082400(ILL6), Potri.008g101600(GA2OX4), Potri.011g057000(ERF9), Potri.002g039100(ERF1), Potri.008g164400(ETR2), Potri.001g166200(JAZ1), and Potri.003g068900(JAZ6), while downregulating the expression levels of Potri.003g080600(ERF5), Potri.015g035800(JAZ3), Potri.005g237900(HVA22), Potri.007g075200(GRAM), and Potri.005g187500(PIN1) (Figure 9D and Figure S3D, Table S5).

## 4. Discussion

The physiological evaluation of *P. simonii* × *P. nigra* revealed dynamic growth responses dependent on stress intensity and duration. Notably, the transient growth stimulation observed at the low concentration (50 mM) at 7 days can be interpreted in the context of hormesis. In this early phase, a subtoxic dose of the stress factor likely acts as a stimulatory signal, actively shaping the plant’s adaptive response and temporarily enhancing physiological vigor before prolonged exposure becomes detrimental. To understand the molecular basis of this adaptation and the severe inhibition caused by high-concentration stress, we explored the underlying transcriptional and metabolic reprogramming.

### 4.1. Plant Hormone Signal Transduction

Abscisic acid (ABA), salicylic acid (SA), jasmonic acid (JA), and ethylene (ET) are key signaling molecules that mediate plant responses to both biotic and abiotic stresses. Generally, ABA primarily regulates abiotic stress responses, as environmental factors such as drought, salinity, low temperature, high temperature, and mechanical damage trigger ABA accumulation [42,43]. In contrast, SA, JA, and ET are central to biotic stress responses [44]. However, plant stress adaptation involves extensive crosstalk among these phytohormone signaling pathways.

ABA has long been recognized for its prominent role in abiotic stress resistance. Under osmotic stresses such as salinity, alkalinity, and drought, ABA maintains water balance by rapidly inducing stomatal closure [45] and promotes longer-term adaptation by regulating stress-responsive genes. Stress conditions induce the expression of ABA biosynthetic genes, driving ABA accumulation [46]. ABA-responsive element-binding proteins (AREBs) and ABA-responsive element-binding factors (ABFs) bind to ABA-responsive elements (ABREs) [47]. Consistently, in *P. simonii* × *P. nigra*, saline-alkaline stress significantly upregulated ABA-responsive genes, leading to substantial ABA accumulation (Figure 9D, Tables S2 and S5). These results suggest that saline-alkaline stress strongly promotes ABA signaling, particularly at high concentrations. ET also plays diverse roles in plant growth, development, and stress responses [48]. ET response factor (ERF) proteins regulate growth and stress adaptation. Overexpression of *VaERF3* in transgenic *Arabidopsis* tolerance to saline-alkaline stress increases proline accumulation while reducing MDA and ROS levels [49]. Similarly, GsERF6 and GsERF71 enhance HCO3- tolerance under alkali stress [50,51]. In this study, the expression levels of ERS and ETR were strongly induced by saline-alkaline stress (Figure 9D, Table S5), and multiple ERF family members showed significant transcriptional changes (Figure 9D, Table S5). KEGG enrichment further confirmed significant enrichment of plant hormone signal transduction across multiple comparisons (Figure 3). Together, these findings suggest that ABA and ET mediate the response of *P. simonii* × *P. nigra* to saline-alkaline stress by activating hormone signaling pathways.

Rather than acting as isolated pathways, plant hormones and metabolic processes form a highly coordinated regulatory network. Our integrated analysis revealed a specific interplay between ABA and JA signaling under saline-alkaline stress in *P. simonii* × *P. nigra*. The rapid accumulation of ABA and JA strongly correlated with the robust upregulation of key signaling components, including Potri.002G125400(ABF2), Potri.008G059200(PP2CA), Potri.003G147300(MYC4), and Potri.019G014300(EIN4) (Figure 7A–D). This upstream hormonal signaling cascade appears to converge on key transcriptional regulators identified in our WGCNA (the ‘pink’ module), specifically the hub transcription factors HB7, MYB108, and NAC055 (Figure 8E). We propose a hierarchical regulatory model, the stress-induced ABA/JA signals putatively activate these master TFs, which subsequently exhibit highly coordinated expression with the extensive metabolic reprogramming observed in our metabolomic data. This includes the upregulation of genes involved in starch and sucrose metabolism (SPS1F, AMY2, BAM1/3/9) to provide carbon skeletons for osmotic adjustment, and the activation of flavonoid biosynthesis genes, Potri.010G213000(CHI1), to enhance antioxidant capacity (Figure 9B, Table S3, Figure 7E). The ABA/JA–transcription factor–metabolic regulatory axis constitutes the core biological insight into the saline-alkaline adaptation of this hybrid poplar.

### 4.2. Accumulation of Osmoregulatory Substances

Plants typically accumulate osmoregulatory substances such as proline, sugars, and betaine to balance extracellular osmotic pressure, thereby maintaining water content and alleviating salt-alkali-induced osmotic stress [52,53]. In *P. simonii* × *P. nigra*, saline-alkaline stress significantly increased the levels of amino acids including proline, tyrosine, phenylalanine, leucine, and isoleucine, as well as sugars such as D-fructose, D-glucose, raffinose, arabinose, and maltotriose. Flavonoids and alkaloids also accumulated in large quantities, including catechins, isoquercitrin, (±)-naringin, trans-chalcone, gentian alkaloids, and caffeoylcholine (Figure 5A,B, Table S2). Previous studies support these findings, particularly recent integrated omics analyses focusing on woody perennials. While foundational studies have shown that overexpressing certain stress-responsive genes in herbaceous models enhances proline accumulation [49,54], our metabolic profiles more closely mirror the conserved long-term adaptive strategies of woody species exposed specifically to NaHCO_3_/Na_2_CO_3_ stress. For instance, recent transcriptomic and metabolomic integrations in *Malus halliana* [55] and rose petals [23] have similarly demonstrated that perennial woody plants actively reroute carbon flux away from primary growth toward the massive biosynthesis of soluble sugars, free amino acids, and flavonoids to maintain osmotic and redox homeostasis. Carbohydrates also play important roles as osmoprotectants. Sucrose mediates phenylalanine, tryptophan, and alkaloid accumulation in *Malus halliana* to maintain osmotic potential [55]. In apples, the s-acyltransferase MdPAT16 promotes sugar accumulation and improves tolerance to salt-alkali-induced osmotic stress [56]. Taken together, these results suggest that *P. simonii* × *P. nigra* maintains osmotic balance under saline-alkaline stress by transcriptionally regulating amino acid, starch/sugar, and secondary metabolic pathways, leading to the accumulation of amino acids, sugars, alkaloids, and flavonoids. This metabolic reprogramming underpins this species’ tolerance to saline-alkaline stress.

Crucially, the massive accumulation of these protective metabolites comes at a significant energetic cost, necessitating a profound resource reallocation. This is strongly supported by the widespread downregulation of genes associated with photosynthesis and cell wall biosynthesis observed under severe stress (Figure 9A). This transcriptional suppression represents a classic growth–defense tradeoff. By downregulating cell wall expansion, the plant actively suspends primary growth, which directly accounts for the pronounced biomass reduction and phenotypic growth arrest observed under the 200 mM treatment (Figure 1D–G). Concurrently, the suppression of photosynthetic light reactions serves as a protective mechanism to prevent the over-reduction of the electron transport chain and minimize excessive ROS generation when the Calvin cycle is restricted. Ultimately, this strategic shutdown of energy-demanding growth processes allows the plant to redirect its limited carbon skeletons and ATP toward the synthesis of crucial survival molecules, including sugars, amino acids, and flavonoids.

### 4.3. Oxidative Stress Response

Plants growing under saline-alkali conditions often experience oxidative stress, which leads to metabolic imbalance and disrupted physiological functions [57]. Oxidative damage results from the excessive accumulation of reactive oxygen species (ROS). To mitigate oxidative damage, plants activate ROS-scavenging systems, including antioxidant enzymes and non-enzymatic antioxidants. Key enzymes include SOD, CAT, POD, glutathione peroxidase (GPX), glutathione reductase (GR), and ascorbate peroxidase (APX), while common non-enzymatic antioxidants include glutathione (GSH), ascorbic acid (ASA), mannitol, flavonoids, anthocyanins, and vitamin E. Together, these defenses limit ROS accumulation and alleviate oxidative stress [5,58]. In this study, both high- and low-concentration saline-alkaline stress increased SOD, POD, and CAT activity in *P. simonii* × *P. nigra* leaves (Figure 2A–C). Metabolomic analysis further showed that flavonoid accumulation increased significantly after 14 days of high-concentration stress (Table S2). This robust antioxidant defense is systematically modulated by the integrated signal-to-adaptation mechanism. As demonstrated by our gene–metabolite correlation networks, the initial hormonal stress signals are highly coordinated with the transcriptional reprogramming of this defense system. Specifically, the hierarchical activation of hormone signaling leads to a strong co-expression linkage between specific structural genes and antioxidant metabolite accumulation. The robust transcriptional activation of key enzymes like Potri.010G213000(CHI1), Potri.005G052200(CFI1), and Potri.010G125400(TKPR2), coupled with the downregulation of repressors like Potri.001G093300(TT2), is strongly associated with driving the metabolic flux toward enhanced flavonoid biosynthesis, resulting in the marked accumulation (through pinocembrin chalcone and eriodictyol) of high-concentration stress, particularly after 14 days (Figure 7E). Critically, the massive accumulation of these specific flavonoids in the leaves likely plays a targeted role in protecting the photosynthetic apparatus. Under prolonged alkaline salt stress spanning 7 to 14 days, carbon assimilation is inherently restricted by osmotic constraints, a state corroborated by the widespread downregulation of photosynthetic genes (Figure 9A). Consequently, chloroplasts become highly susceptible to over-reduction of the electron transport chain, leading to severe electron leakage and photoinhibition. In this context, the accumulated flavonoids can function synergistically to mitigate this damage. They not only act as an optical filter to shield the thylakoids from excess excitation energy but also serve as potent scavengers to detoxify the reactive oxygen species leaking from the impaired chloroplasts, thereby preserving cellular integrity under prolonged stress. These findings indicate that saline-alkaline stress triggers oxidative stress responses in *P. simonii* × *P. nigra* by enhancing antioxidant enzyme activity and promoting the accumulation of antioxidant metabolites, thereby reducing ROS damage.

### 4.4. Transcription Factors

During salt-alkali stress signaling, TFs act as key regulators that link external stimuli to downstream stress-responsive genes binding to specific cis-regulatory elements [11,59]. In *P. simonii* × *P. nigra*, saline-alkaline stress significantly altered the expression of several TF families, including WRKY, MYB, AP2/ERF, bHLH, NAC, and C2H2 (Figure 8E and 9D). Previous studies have highlighted their central roles in stress adaptation. For example, ectopic expression of *SlWRKY28* in *Populus davidiana × P. bolleana* enhanced tolerance to saline-alkaline stress [60]. While investigations in herbaceous crops like sugar beet and soybean provide valuable baseline data regarding WRKY and MYB functions [61,62,63], comparative transcriptomic studies in woody species offer a more direct and relevant context for our findings. Consistent with our WGCNA results, recent omics profiling of woody plants such as *Populus davidiana × P. bolleana* [60], *Malus halliana* [12], and *Xanthoceras sorbifolia* [11] subjected to alkaline stress frequently identifies the AP2/ERF, NAC, and MYB families as the core hierarchical regulators driving stress adaptation. The robust co-expression of PsnHB7, PsnMYB108, and PsnNAC055 observed in our ‘pink’ module further confirms that perennial trees rely on a highly specialized, woody-specific network of these stress-responsive hub genes to orchestrate long-term adaptation to alkaline soil environments. Similarly, ThNAC13 enhanced salt and osmotic tolerance in *Tamarix* and *Arabidopsis* by enhancing ROS scavenging and improving osmotic regulation [64]. Collectively, these previous findings, together with our results, suggest that multiple TF families, including WRKY, MYB, ERF, and NAC, contribute to saline-alkaline stress tolerance through dynamic regulation of the stress-responsive gene expression of *P. simonii* × *P. nigra*.

**Figure 10:**
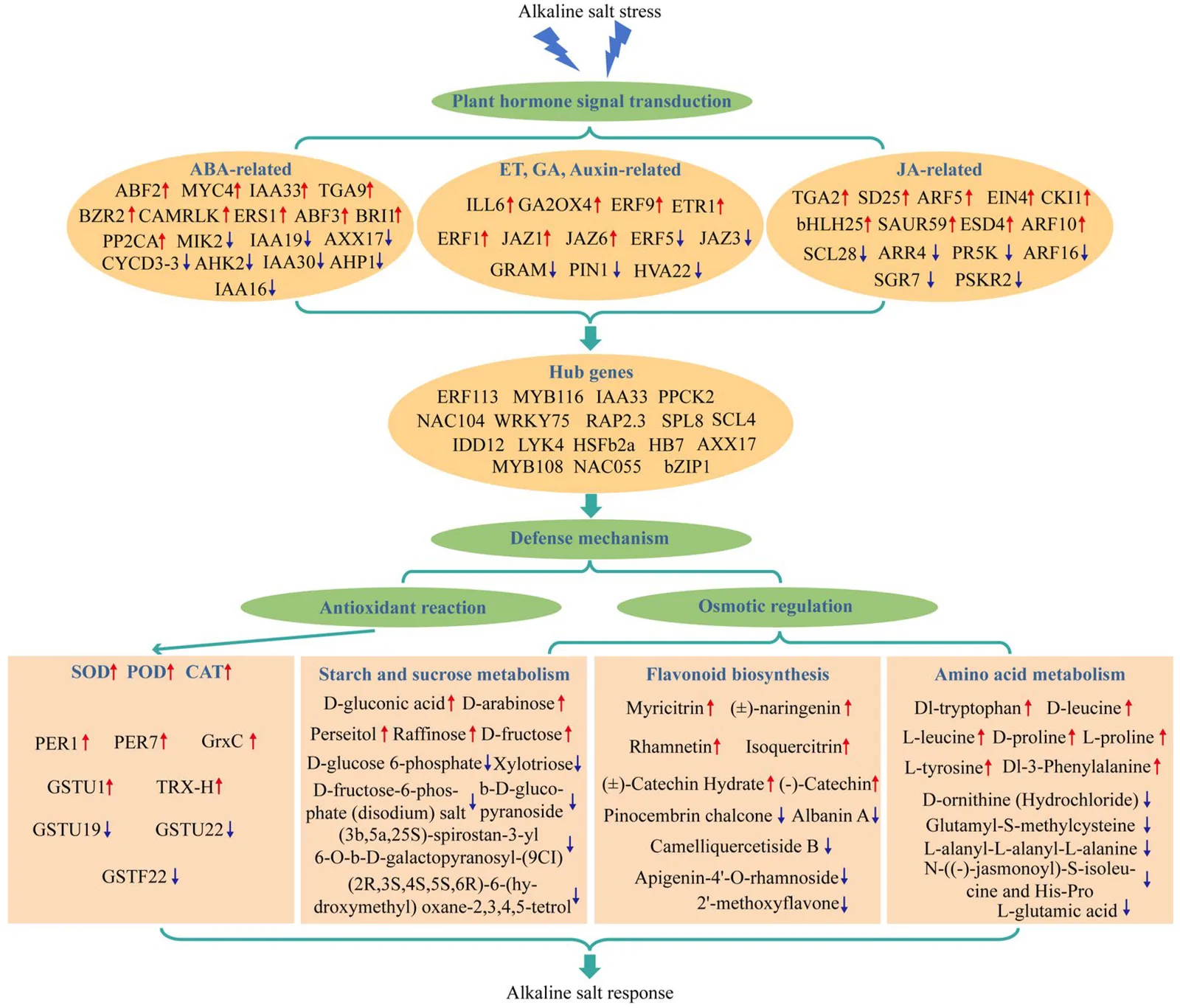
Schematic diagram of the physiological and molecular mechanisms underlying the response of *P. simonii* × *P. nigra* to saline-alkaline stress. Red arrows indicate the upregulation of genes/metabolites, while blue arrows indicate the downregulation of genes/metabolites.

Based on the integrated analysis of our multi-omics data, we propose a hierarchical regulatory network elucidating the physiological and molecular adaptation of *P. simonii* × *P. nigra* to saline-alkaline stress (Figure 10). The perception of alkaline salt stress triggers a coordinated hormonal response, primarily mediated by the ABA, ET, and JA pathways. Rather than acting independently, these upstream signals converge to activate a core regulatory module composed of key stress-responsive transcription factors, notably members of the AP2/ERF, MYB, and NAC families. These master switches, in turn, are implicated in orchestrating a systemic defense response characterized by a distinct resource reallocation strategy. By actively suppressing energy-demanding primary growth, the plant redirects critical carbon and nitrogen fluxes toward two primary survival mechanisms: the enhancement of the enzymatic antioxidant system to mitigate photo-oxidative damage, and the profound reprogramming of osmotic regulatory metabolism. This targeted metabolic shift drives the massive biosynthesis of protective soluble sugars, free amino acids, and antioxidant flavonoids. Ultimately, the precise synchronization across transcriptional regulation and metabolic flux constitutes a robust systemic strategy, enabling this perennial species to reestablish cellular homeostasis in highly degraded alkaline soils. While our findings provide valuable molecular insights into alkaline salt adaptation, certain limitations of the current study must be acknowledged. Although our experimental pots contained 400 g of substrate to provide adequate rooting space, the greenhouse environment inevitably differs from the natural soil dynamics of the Songnen Plain. Pot-based systems can introduce edge effects and lack the complex pH buffering capacity, nutrient cycling, and microbial interactions present in natural saline-alkaline fields. Furthermore, while the WGCNA and O2PLS models successfully identify hub transcription factors exhibiting highly coordinated expression with metabolic reprogramming, these networks remain predictive. Conclusive establishment of direct biochemical causality will require future in vivo functional validations such as targeted gene overexpression or CRISPR-Cas9-mediated knockouts, alongside long-term field trials to fully elucidate the ecological adaptation of *P. simonii* × *P. nigra*.

## 5. Conclusions

This investigation reveals that saline-alkaline stress substantially inhibits the growth of *P. simonii* × *P. nigra* growth while undergoing extensive physiological, transcriptomic, and metabolomic changes. High-concentration stress (200 mM) induces oxidative damage, as evidenced by increased malondialdehyde (MDA) content and enhanced antioxidant enzyme activities, accompanied by the accumulation of osmoregulatory compounds. Combined transcriptomic and metabolomic analyses demonstrate extensive molecular reprogramming, with significant enrichment of genes and metabolites involved in plant hormone signaling, phenylpropanoid and flavonoid biosynthesis, starch and sucrose metabolism, and amino acid metabolism. WGCNA analysis identifies co-expression modules and hub genes closely associated with saline-alkaline stress responses, highlighting the important regulatory roles of transcription factors, including MYB, AP2/ERF, NAC, bZIP, ARF, and WRKY. Genes involved in starch and sucrose metabolism, raffinose metabolism, amino acid biosynthesis, and antioxidant metabolism were markedly regulated, reflecting coordinated metabolic reprogramming during stress adaptation. These results provide new insights into the physiological and molecular mechanisms underlying saline-alkaline stress adaptation in *P. simonii* × *P. nigra* and provide a valuable foundation for the genetic improvement of stress-tolerant poplar germplasm.

## Data Availability

The RNA-seq data under alkaline stress can be found under accession number GSE285903.

## Supplementary Materials

The following supporting information can be downloaded at https://www.mdpi.com/article/10.3390/biology15181656/s1, Figure S1: Expression patterns and KEGG analysis of DEGs in the “brown” and “lightgreen” module of WGCNA; Figure S2: Validation of nine hub genes’ expression data from RNA-seq by RT-qPCR; Figure S3: Pathway mapping of DEGs in CK-14d vs. L-14d. Table S1: The gene-specific primers used in this study; Table S2: Detailed information of metabolites; Table S3: The detailed information of starch and sugar degradation; Table S4: The detailed information of antioxidant reaction and plant hormone signal transduction; Table S5: The detailed information of raffinose metabolism.
